# The Essential Role of OmpR in *Acidithiobacillus caldus* Adapting to the High Osmolarity and Its Regulation on the Tetrathionate-Metabolic Pathway

**DOI:** 10.3390/microorganisms11010035

**Published:** 2022-12-22

**Authors:** Linxu Chen, Xiao Liu, Chang Gao, Yanan Guan, Jianqiang Lin, Xiangmei Liu, Xin Pang

**Affiliations:** State Key Laboratory of Microbial Technology, Shandong University, Qingdao 266237, China

**Keywords:** *Acidithiobacillus caldus*, osmotic regulation, EnvZ/OmpR, sulfur oxidation, S_4_I pathway

## Abstract

*Acidithiobacillus* spp. are prevalent in acid mine drainage, and they have been widely used in biomining for extracting nonferrous metals from ores. The osmotic stress generated by elevated concentrations of inorganic ions is a severe challenge for the growth of *Acidithiobacillus* spp. in the bioleaching process; however, the adaptation mechanism of these bacteria to high osmotic pressure remains unclear. In this study, bioinformatics analysis indicated that the osmotic stress response two-component system EnvZ-OmpR is widely distributed in *Acidithiobacillus* spp., while OmpRs from *Acidithiobacillus* spp. exhibited a far more evolutionary relationship with the well-studied OmpRs in *E. coli* and *Salmonella typhimurium*. The growth measurement of an *Acidithiobacillus caldus* (*A. caldus*) *ompR*-knockout strain demonstrated that OmpR is essential in the adaptation of this bacterium to high osmotic stress. The overall impact of OmpR on the various metabolic and regulatory systems of *A. caldus* was revealed by transcriptome analysis. The OmpR binding sequences of differentially expressed genes (DEGs) were predicted, and the OmpR box motif in *A. caldus* was analysed. The direct and negative regulation of EnvZ-OmpR on the tetrathionate-metabolic (*tetH*) cluster in *A. caldus* was discovered for the first time, and a co-regulation mode mediated by EnvZ-OmpR and RsrS-RsrR for the tetrathionate intermediate thiosulfate-oxidizing (S_4_I) pathway in this microorganism was proposed. This study reveals that EnvZ-OmpR is an indispensable regulatory system for the ability of *A. caldus* to cope with high osmotic stress and the significance of EnvZ-OmpR on the regulation of sulfur metabolism in *A. caldus* adapting to the high-salt environment.

## 1. Introduction

*Acidithiobacillus*, a genus of acidophilic and chemoautotrophic sulfur-and/or iron- oxidizing bacteria, is prevalent in sulfur-containing acidic niches [1,2].These bacteria could promote the geobiochemical circulation of elements and the release of heavy metals in mining-associated habitats [1]. Members in this genus have been widely applied in the bioleaching industry to recover nonferrous metals (e.g., copper, gold, or uranium) from different ores [3]. All strains of *Acidithiobacillus* possess the capacity to oxidize various reduced inorganic sulfur compounds such as tetrathionate (S_4_O_6_^2−^), thiosulfate (S_2_O_3_^2−^), sulfite (SO_3_^2−^), sulfide (S^2−^) and elemental sulfur (S^0^) [2]. Additionally, some species, including *A. ferrooxidans*, *A. ferrivorans*, *A. ferriphilus* and *A. ferridurans*, can oxidize ferrous iron for autotrophic growth [4,5,6]. Extensive studies have been conducted on the three typical species of *Acidithiobacillus*: *A. ferrooxidans*, *A. thiooxidans*, and *A. caldus* [7,8,9,10]. 

In the natural mine habitats, *Acidithiobacillus* spp. can metabolize the sulfur and ferrous iron deposited in minerals, generating the ion-enriched environments. For example, a sulfate concentration up to 7.9 M was detected in the water of the abandoned Richmond mine at Iron Mountain, California [11]. Metal ions, such as Fe^3+^, Cu^2+^, and Zn^2+^, are released from ores as the bioleaching reaction progresses. Dissolved inorganic ions exert multiple pressures on the growth of these autotrophic bacteria, including the following: the low acidity caused by the increase of sulfuric acid, the high oxidation potential generated by increasing ferric iron, the toxicity of heavy metals, and the osmotic stress caused by high concentrations of various ions.

Research has shown that inorganic ions and osmotic pressure are severe environmental factors for the growth of *Acidithiobacillus* spp. as well as the efficiency of the bioleaching of minerals. It has been reported that the inhibitory effect of Na_2_SO_4_ or K_2_SO_4_ is stronger than that of CuSO_4_ or ZnSO_4_, and weaker than that of KCl or NaCl [12]. Iron-oxidizing bacteria such as *A. ferrooxidans* and *Leptospirillum ferriphilum* are highly sensitive to the level of Cl^−^ because of the inhibitory effect of chloride on ferrous iron oxidation [13,14]. The sulfur oxidizer *A. caldus* has a much higher tolerance of NaCl stress (1.0 M NaCl) than *A. ferrooxidans* (0.12 M) [15]. Conversely, other ions such as SO_4_^2−^, Na^+^, and K^+^, have little effect beyond their influence on osmotic stress [16,17]. Thus, an elevated ion concentration will cause osmotic stress, which could restrict the growth of these acidophilic bacteria, and consequently, the bioleaching of sulfide minerals. The discoveries of compatible solutes (trehalose, glucose and proline) in several species of acidophilic bacteria and up-regulation of some proteins in the presence of NaCl stress in *A. caldus*, indicate that *Acidithiobacillus* spp. might employ different strategies to respond to osmotic stress [15,18]. 

The EnvZ-OmpR two-component system is well known to mediate osmotic stress response in bacteria [19,20]. In *Escherichia coli*, the histidine kinase EnvZ monitors environmental osmolarity and is autophosphorylated at high osmolarity, the phosphoryl group is then transferred to the response regulator OmpR [19,21]. Phosphorylated OmpR (OmpR-P) binds to the promoter regions of outer membrane porin genes *ompF* and *ompC* and regulates their expression according to the cellular OmpR-P level [22,23]. By the regulation of EnvZ-OmpR on OmpC and OmpF, *E. coli* could govern the outer membrane permeability and adapt to the osmotic stress [24,25,26]. In addition to osmoregulation, application of the EnvZ-OmpR system has been extended to the fatty acid receptor, peptide permease, flagella acid shock and stationary-phase acid tolerance response regulation [27,28,29]. Thus, it has become apparent that OmpR plays a central regulatory role in the expression of a large number of genes, including small RNAs [30], in response to various signals from the environment in a wide variety of organisms. 

Genomic information implied that there may be EnvZ and OmpR analogs in *Acidithiobacillus* spp. [31,32,33]. Moreover, the role of EnvZ-OmpR in osmoregulation and the EnvZ-OmpR-mediated regulatory strategy in *Acidithiobacillus* spp. are still unclear due to significant differences in *Acidithiobacillus* spp. and heterotrophic bacteria with regard to energy metabolism, biosynthesis and growth environment. In this study, we analyzed the distribution of EnvZ-OmpR system in *Acidithiobacillus* spp., determined the function of this system in *A. caldus* responding to the high osmotic pressure, explored and identified the genes regulated by EnvZ-OmpR. Finally, an EnvZ-OmpR-mediated novel regulatory mode for the sulfur oxidation and osmoregulation was proposed in *A. caldus*. 

## 2. Materials and Methods

### 2.1. Bacterial Strains and Growth Conditions

The bacterial strains and plasmids used in this study are listed in Table 1. The strain *A. caldus* MTH-04 has been deposited in the China General Microbiological Culture Collection Center (CGMCC) under accession number CGMCC 1.15711. Starkey -S^0^ liquid medium and solid Starkey-Na_2_S_2_O_3_ plates for *A. caldus* growth were prepared as reported previously [34]. Chloromycetin, kanamycin, and streptomycin (Sigma, St. Louis, MO, USA) were added to a final concentration of 34, 100, and 100 μg/mL in LB medium, and 60, 100, and 100 μg/mL in both liquid and solid Starkey media, respectively. The culturing conditions were 37 °C and 200 r/min for *E. coli* and 40 °C and 150 r/min for *A. caldus* MTH-04. The cultivation method and cell measurement for *A. caldus* were performed according to previously reported methods [35]. 

### 2.2. Construction of the ompR Knockout Strain of A. caldus 

First, homologous 5′ and 3′ flanking regions of *ompR* were amplified using primers P1-F/R and P2-F/R, respectively. The products were further digested and ligated into the vector pSDUDI, generating the suicide plasmid pSDUDI-ΔompR. Second, this constructed plasmid was conjugated from *E. coli* SM10 to *A. caldus* according to a published protocol [38]. Single crossovers were selected on kanamycin selective Starkey-Na_2_S_2_O_3_ solid plates and confirmed by colony PCR using primers oriT-F/R. Third, the I-Sce I-expressing plasmid (pSDU1-I-Sce I) was conjugated into single crossovers to facilitate the second crossover and generate mutants. Finally, the *ompR* knockout strains were screened by PCR using two sets of primers, ompR-F/R and IHA-F/R, specific for the targeted *ompR* gene and the interior regions of the two homologous arms, respectively. Identification of the *ompR-*knockout strain was performed by PCR using purified genomic DNA (TIANamp Bacteria DNA kit, Tiangen, China) as the templates with three sets of primers (ompR-F/R, IHA-F/R, and LHA-F/R). The PCR fragments amplified by primers LHA-F/R specific for the lateral regions of the two homologous arms were sequenced for verification. Primers used in the construction of *A. caldus ompR*-knockout strain were listed in Table 2.

### 2.3. RNA Extraction and Transcriptional Analysis

*A. caldus* wild-type and mutant strains were cultivated to the 6th day in S^0^ media. Na_2_SO_4_ was added to the cultures at a final concentration of 0.3 M. After an additional 3-h-cultivation, the cultures were filtered through the filter paper to remove the sulfur granules, and all cells were collected by centrifugation at 8000× *g* for 5 min. Preparation of RNA for RNA-seq was according to [39], and the subsequent construction of cDNA libraries and RNA-seq were performed by Novogene (Tianjin, China) [39]. Genes with an adjusted *p* value ≤ 0.05, FDR (false discovery rate) ≤ 0.05 and fold change (FC) ≥ 1.5 were selected as DEGs. 

Real-Time Quantitative PCR (RT-qPCR) was used to confirm the DEGs detected by RNA-seq. All RT-qPCR reactions in this study were performed with three biological replicates. The experimental method and statistical approach are performed according the previous report in [39]. Genes with FC ≥ 1.5, *p* ≤ 0.05 and FC ≤ 0.67, *p* ≤ 0.05 were considered significantly up-regulated and down-regulated, respectively. The standard deviation (SD) value was calculated by using the Origin software, and the *p* value was determined by an unpaired *t*-test using GraphPad Prism software. A Bland-Altman limit of an agreement (LOA) plot was used to carry out agreement comparison of the results from RNA-seq and RT-qPCR. Primers used in RT-qPCR were listed in Table S1.

### 2.4. Expression and Purification of the OmpR Protein

The *ompR* gene of *A. caldus* MTH-04 was amplified with primers ompR_02628_-F and ompR_02628_-R (Table 2). The purified PCR product and pET-28a were digested with BamH I and Hind III, and the generated products were ligated together to obtain plasmid pET-28a-ompR. The sequences of the inserted *ompR* gene were verified by sequencing, and pET-28a-ompR with the correct sequences was transformed into *E. coli* BL21 (DE3). The recombinant OmpR was purified with HisTrap HP column (GE Healthcare, Chicago, IL, USA). The concentration of purified OmpR was determined using the Bradford assay. 

### 2.5. Isothermal Titration Calorimetry (ITC)

To determine the *Kd* (dissociation constant) value and thermodynamic parameters of the interaction between OmpR and predicted OmpR boxes within the *tetH* promoter region of *A. caldus*, OmpR was titrated with the DNA sequence using a MicroCal iTC200 system (GE Healthcare). The purified protein was thoroughly dialyzed at 4 °C against TE buffer (100 mM Tris-HCl, 50 mM EDTA [pH 8.0]). The protein solution (24 μM) was titrated at 30 °C with a double-stranded DNA (dsDNA) solution containing 2 μM OmpR box fragments, with 19 injections of 2 μL after the first injection of 0.5 μL and a time interval of 120 s between injections. The heat effects from a blank experiment (injection of OmpR into the TE buffer) were subtracted before the titration curves were fit to a nonlinear least-squares function. From these curve fits, *Kd*, the change in binding enthalpy (Δ*H*), and the binding stoichiometry were determined. The change in Gibbs free energy (Δ*G*) and the change in entropy (Δ*S*) were calculated using the equations Δ*G* = *RT*ln*Kd* and Δ*G* = Δ*H − T*Δ*S*, where *R* is the universal molar gas constant and *T* is the temperature (in kelvins). DNA fragments were obtained by PCR amplification using the different sets of primers listed in Table 2.

### 2.6. Electrophoretic Mobility Shift (EMSA) Assays

The G360 and T360 fragments were obtained using primer-pairs G360-F/-R and T360-F/-R, respectively. The generated fragments were purified using QIAquick Gel Extraction Kit (Qiagen Corp., Hilden, Germany) and concentrated using Amicon Ultra-15 mL, 3 kDa Centrifugal Filter Unit (Millipore Corp., Burlington, MA, USA). EMSA assays were performed as described previously [35].

## 3. Results

### 3.1. Sequence Analysis and Comparison of OmpRs in Acidithiobacillus *spp.*

**Figure 1:**
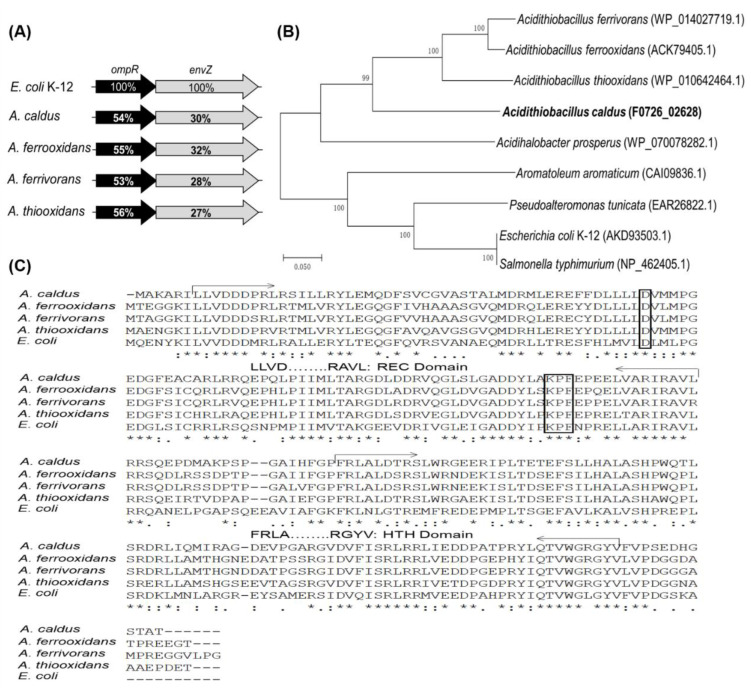
Bioinformatic analysis of OmpRs. (**A**) The ompR-envZ operons from *Acidithiobacillus* spp. The percent identities between protein sequences are indicated by values shown in the genes. Abbreviations: OmpR: osmolarity response regulator; EnvZ: osmolarity sensor protein. Accession numbers (GenBank) for these proteins: *A. caldus* MTH-04, OmpR (F0726_02628), EnvZ (F0726_02627); *A. ferrooxidans* ATCC 23270, OmpR (ACK79405.1), EnvZ (ACK79033.1); *A. ferrivorans* SS3, OmpR (WP_014027719.1), EnvZ (WP_014027720.1); *A. thiooxidans*, OmpR (WP_010642464.1), EnvZ (WP_031575473.1). (**B**) A phylogenetic tree of several OmpRs from *Acidithiobacillus* spp. and other bacteria. CLUSTALX (version 1.81) and MEGA 4.0 with bootstrapping (1000 replicates) were used for phylogenetic tree construction. The protein id or locus_tag of each OmpR is present in parentheses. (**C**) Amino acid sequence alignment of OmpRs from *Acidithiobacillus* spp. and *E. coli* K12. Identical and similar amino acids are indicated. The typical REC and HTH domains are characterized and marked in the sequence. The predicted phosphorylation site (D) and dimerization interface (KPF) are shown in boxes.

Annotations of published genome sequences for the *Acidithiobacillus* genus revealed the presence of OmpR and EnvZ homologues and the existence of *ompR-envZ* operons (Figure 1A). Comparison of amino acid sequences showed that OmpR and EnvZ in *Acidithiobacillus* are distantly related to the respective protein in *E. coli*. OmpRs and EnvZs from *Acidithiobacillus* show approximately 50% and 30% identities to the proteins in *E. coli*, respectively. An unrooted phylogenetic tree was constructed for the predicted OmpRs from some *Acidithiobacillus* strains and several other identified OmpR homologues (Figure 1B). OmpRs from *Acidithiobacillus* spp. exhibit a close relationship between *Acidithiobacillus* species, but a far evolutionary relationship with the well-studied OmpRs in *E. coli* and *S. typhimurium*. By analysing the OmpRs from *Acidithiobacillus* spp. and *E. coli* K12, OmpRs in acidophilic autotrophic *Acidithiobacillus* strains were found to carry the typical signal receiver (REC) and helix-turn-helix (HTH) domains responsible for receiving the phosphoryl group from their cognate EnvZs and binding to regulatory sequences, respectively (Figure 1C). The phosphorylation site (D) and dimerization interface (KPF) in the REC domain are also predicted to be present in the OmpRs from *Acidithiobacillus* (Figure 1C). The presence of a similar *omp*R-*env*Z operon structure, and the discovery of the typical domains and conserved amino acid residues among OmpRs indicated potential similar biological functions of the EnvZ-OmpR system in the acidophilic autotrophic *Acidithiobacillus* spp. and in *E. coli*. 

### 3.2. Construction of the A. caldus ompR-Knockout Strain

**Figure 2:**
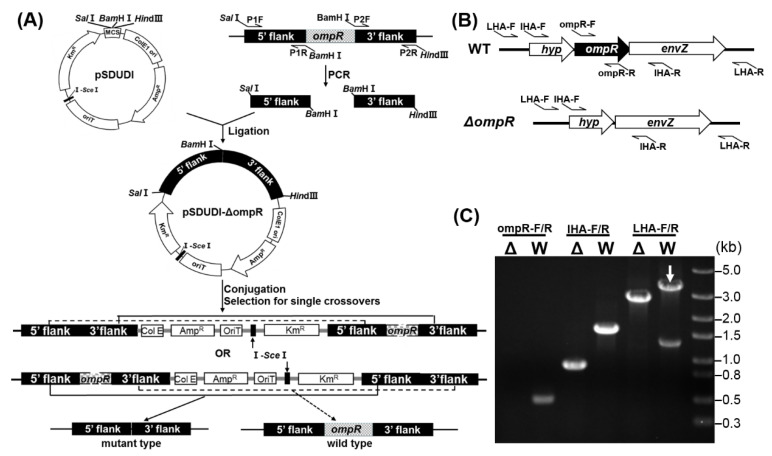
The construction process of the *A. caldus* ompR knockout strain. (**A**) The markerless gene knockout strategy in *A. caldus*. (**B**) Diagram of three sets of primers (ompR-F/R, IHA-F/R and LHA-F/R) specific for ompR, the upstream and downstream homologous arms (UHA and DHA) and the sequences outside of homologous arms, respectively. (**C**) PCR amplification of the genomic DNA of wild-type (W) and mutant (Δ) *A. caldus* MTH-04 using primers ompR-F/R, IHA-F/R and LHA-F/R.

The *ompR* gene in *A. caldus* MTH-04 was successfully deleted using the “in–out” markerless gene knockout strategy developed by our laboratory [35]. As shown in Figure 2A, the “in” step was achieved with the suicide plasmid pSDUDI-ΔompR containing two homologous arms located upstream and downstream of the *ompR* gene. The suicide plasmid integrates into the *A. caldus* genome via allelic exchange, resulting in the generation of a single crossover mutant. The “out” step was performed by introducing plasmid pSDU1-I-SceI expressing the I-SceI endonuclease. The enzyme produces a double-stranded break (DSB) at the I-SceI site in the genome of the single crossover mutant. The DSB stimulates a second allelic exchange, producing a mutant or a wild-type sequence. The *ΔompR* strain was confirmed by PCR using the different sets of primers (Figure 2B,C). No band was amplified from the *ompR* knockout strain when using primers ompR-F/R specific for the targeted gene *ompR*. A smaller fragment was obtained from the mutant using primers UHA-F/DHA-R specific for the homologous arms, and a 2.7 kb fragment was amplified from *ΔompR* compared to a 3.4 kb fragment from the wild-type using primers LHA-F/R specific for the lateral regions of the two homologous arms. Finally, the 2.7 kb PCR fragment amplified from *ΔompR* was sequenced for verification. The results indicated that the *ΔompR* strain carries a markerless in-frame *ompR* mutation with deletion of a 720 bp sequence from the start (ATG) to the stop (TGA) codes. 

### 3.3. The Influence of Ion Concentration on the Growth of A. caldus and its ompR Mutant

**Figure 3:**
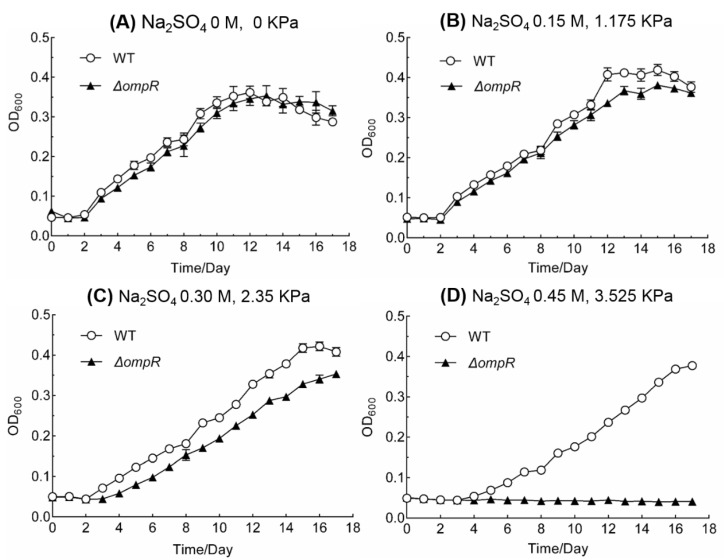
Growth curves of ΔompR and wild-type strains of *A. caldus* MTH-04 at different concentrations of sodium sulfate in S0 medium (OD600, optical density at 600 nm). The two numbers (×× M, ××× KPa) stand for the concentration of added salts and the generated osmotic pressure by these added compounds, respectively. Experiments were performed in triplicate. Each data point represents the arithmetic mean value of the three parallel experimental groups. The error bars indicate standard deviations.

To investigate the effect of osmotic stress on *A. caldus* cells, we examined the growth of *ΔompR* and wild-type strains in liquid starkey-S^0^ medium with different concentrations of inorganic ions. When sodium sulfate was used as the reagent for generating osmotic pressure, the mutant exhibited an obvious disadvantage in growth compared to that of the wild-type strain with an increase in the concentration. The *ΔompR* strain displayed a slightly weaker growth pattern compared to that of the wild-type strain at Na_2_SO_4_ concentration of 0 and 0.15 M (Figure 3A,B). When the concentration of Na_2_SO_4_ was increased to 0.30 M, the growth of the mutant was obviously suppressed compared to that of the wild-type strain (Figure 3C). *ΔompR* was unable to grow when the concentration of Na_2_SO_4_ reached 0.45 M (Figure 3D). Similar phenomena were observed when K_2_SO_4_ was used to test the sensitivity of the mutant to osmotic stress (Figure S1). Although the growth of *ΔompR* was suppressed by NaCl at concentrations of 0.15, 0.30 or 0.45 M, the mutant could grow at 0.45 M (Figure S1). Therefore, the poor growth of *ΔompR* in the presence of elevated concentrations of inorganic salts, indicated the critical role of OmpR in *A. caldus* adaptation to the osmotic stress caused by inorganic ions.

### 3.4. The Influence of OmpR Absence on the Transcriptome Profile of A. caldus under Osmotic Stress

Total RNA from the wild-type and mutant strains under an osmotic pressure of 0.3 M Na_2_SO_4_ was extracted to carry out RNA-seq for detecting DEGs. A total of 109 DEGs, including 75 down-regulated and 34 up-regulated DEGs in the OmpR knockout strain (see Supplementary Table S2), were selected using the criteria described in the Methods Section 2.3. Reverse transcription-quantitative PCR (RT-qPCR) using the primers listed in supplementary Table S1 was performed on 35 of the 109 DEGs; fold changes were calculated (Table S2), and these fold change values based on RT-qPCR were compared to those of RNA-seq. The results indicated that among the 35 genes, data for 33 were within the 95% confidence interval (see Supplementary Figure S2), suggesting high consistency between the results generated by RNA-seq and RT-qPCR. Thus, the fold changes of genes determined by RNA-seq are reliable data.

**Figure 4:**
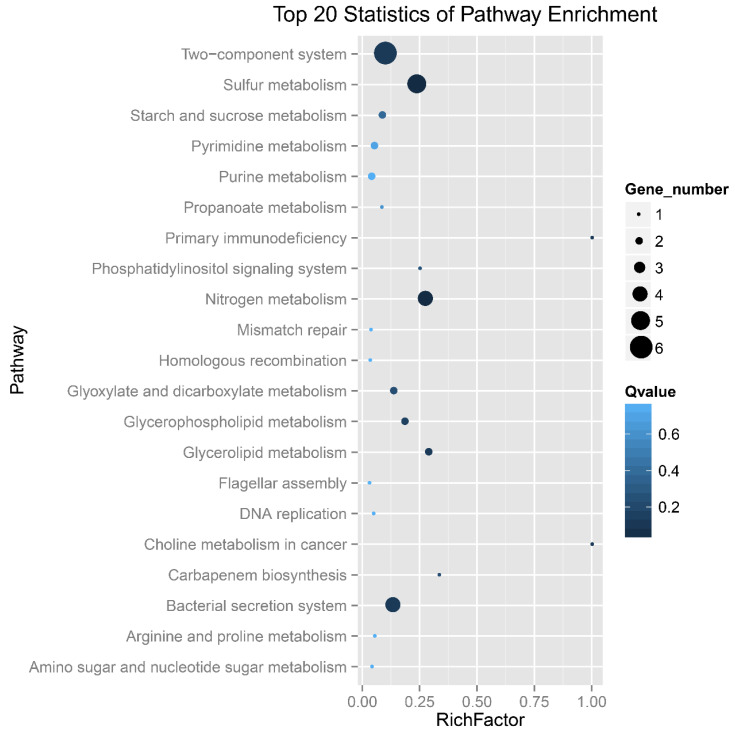
Scatter plot of KEGG pathway enrichment analysis of DEGs in *ΔompR* compared to wild-type *A. caldus* MTH-04. Gene number: number of DEGs mapped to a certain pathway. Enrichment factor: The ratio of the number of DEGs mapped to a certain pathway to the total number of genes mapped to this pathway. The Q-value is the corrected *p*-value ranging from 0–1 and a lower value indicates greater intensiveness.

The 109 DEGs are mainly involved in the metabolic processes of sulfur, nitrogen and carbon, membrane and channel proteins, conjugal transfer system and DNA modification system (Table S2 and Figure 4). Deletion of *ompR* resulted in up-regulation of *tetH* and *tqo* and down-regulation of *soxY* (F0726_02556), *soxZ* (F0726_02557), *soxB* (F0726_02558), *soxA* (F0726_02562) and *sdo*, indicating the influence of EnvZ-OmpR on sulfur oxidation in *A. caldus*. Many channels and membrane proteins showed obvious down-regulation in the *ompR* mutant, such as the TonB-dependent receptor (F0726_03003), ABC-type Mn^2+^/Zn^2+^ transport system (F0726_00324, 00325 and 00326) and RND efflux system(F0726_01023), suggesting regulation of membrane permeability by OmpR. Expression of flagellar synthesis genes (F0726_01512, 02316 and 02317) was down-regulated in the mutant, implying the influence of EnvZ-OmpR on cell motility in *A. caldus* adaptation to osmotic stress. In addition, the absence of OmpR resulted in down-regulation of the restriction-modification system, transposases, regulators and cyclic di-GMP metabolism-related proteins. The majority of DEGs are down-regulated in *ΔompR*, indicating that the mutant had to reduce metabolism, membrane permeability and cell motility to adapt to the osmotic pressure. Therefore, EnvZ-OmpR played a global regulatory role in the osmoregulation of *A. caldus*.

### 3.5. OmpR Binding Sequences in A. caldus

**Figure 5:**
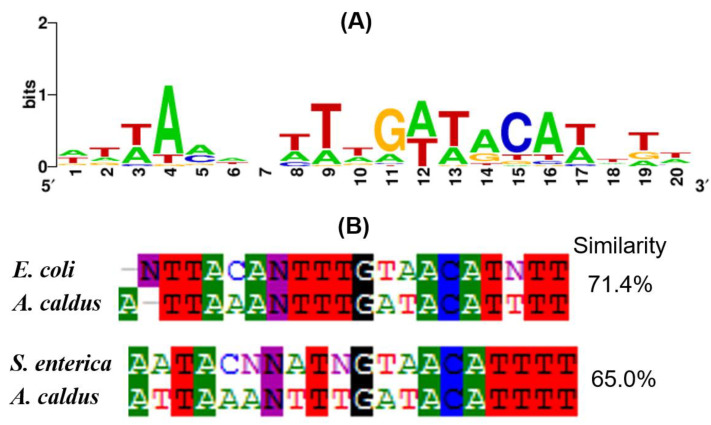
Analysis of the OmpR box motif from *A. caldus*. (**A**) The motif of *A. caldus*; (**B**) alignment of OmpR binding motifs.

To discover OmpR binding sequences (OmpR boxes) in *A. caldus*, the 700 bp sequence upstream of the DEGs were used for scanning OmpR boxes using the matrix-scan program at http://rsat.ulb.ac.be/rsat, with a cutoff score value of 7. As shown in Table 3, OmpR consensus-like sequences were discovered upstream of 25 DEGs, including 10 up-regulated and 15 down-regulated genes. Furthermore, these predicted OmpR binding sequences in *A. caldus* MTH-04 were used to create the logo motif of OmpR boxes (Figure 5A). The OmpR-binding sequence motif from *A. caldus* displayed 71.4% and 65.0% similarity to that from *E. coli* and *Salmonella enterica* (*S. enterica*), respectively (Figure 5B). Overall, the discovery of OmpR binding sequences upstream of the DEGs and the high similarities of OmpR box motifs between *A. caldus* and other bacteria, indicate the conservation of OmpR during its evolution in these chemoautotrophic and acidophilic bacteria.

### 3.6. The Binding Ability of OmpR to the tetH Promoter Fragment

**Figure 6:**
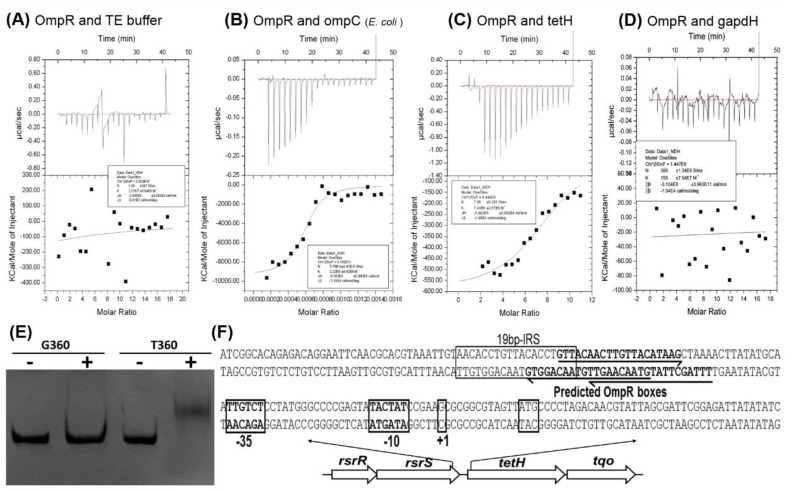
The binding affinity of OmpR to the tetH promoter region. (**A**–**D**) are the results of ITC experiments titrating *A. caldus* OmpR with the TE buffer, ompC fragment, tetH fragmentand gapdH fragment, respectively. (**E**) The results of EMSA to display the binding of OmpR to the *tetH* promoter region. (**F**) Schematic diagram of the regulatory elements in the *tetH* promoter region. G360, a 360 bp fragment from the gapdH gene used as a control; T360, amplified 360 bp fragment upstream of the *tetH* gene.

The two co-transcribed genes *tetH* (encoding tetrathionate hydrolase) and *tqo* (encoding thiosulfate: quinol oxidoreductase), combined with a two-component system (RsrR and RsrS), are arranged in a cluster in *A. caldus* [40]. Up-regulation of *tetH* and tqo in *ΔompR* and the predicted OmpR boxes at upstream of the *tetH* gene suggest a negative and direct regulation of *tetH* by OmpR (Table S2 and Table 3). To confirm this, assays, including isothermal titration calorimetry (ITC) and electrophoretic mobility shift assay (EMSA), were performed to confirm binding between OmpR and the *tetH* promoter sequence. In the ITC analysis, no obvious differences in the integration effect were found between OmpR and the TE buffer (Figure 6A). An obvious reaction signal was detected using the *E. coli* ompC fragment containing the conserved OmpR box (Figure 6B), indicating that OmpR from *A. caldus* is able to bind to the OmpR box sequence. OmpR showed a significant integration effect with the tetH fragment but not the gapdH fragment from *A. caldus* (Figure 6C,D), showing the ability of OmpR to bind to the *tetH* promoter region. Furthermore, the binding ability was confirmed by EMSA. The expected gel shift of OmpRwith the tetH fragment and no shift of OmpRwith the gapdH fragment were observed (Figure 6E). The consistent results from ITC and EMSA indicated that OmpR is able to bind directly to the *tetH* promoter region to achieve negative regulation of tetrathionate metabolism of *A. caldus* (Figure 6F).

## 4. Discussion

Our study revealed that the EnvZ-OmpR system is an essential osmoregulation mechanism for *Acidithiobacillus* spp. to adapt to a high-salt environment. The discovery of EnvZ-OmpR homologues and the *ompR-envZ* operon in different species of *Acidithiobacillus* indicate the presence of the EnvZ-OmpR system in these chemoautotrophic sulfur-oxidizing bacteria (Figure 1A). While the OmpRs from *Acidithiobacillus* strains are distantly related to those from *E. coli* and *S. typhimurium* (Figure 1B), the typical domains and conserved amino acid residues of OmpRs in these acidophilic autotrophic bacteria are almost identical to that of *E. coli*. The significant inhibitory effect of high ion concentrations on the growth of *ΔompR* demonstrates the essential role of OmpR for *A. caldus* to adapt to high osmotic stress (Figure 3). Overall, the prevalence and conservation of EnvZ-OmpR in *Acidithiobacillus* spp. as well as the determination of the indispensable role of OmpR in the growth of *A. caldus* at high concentrations of inorganic salts suggest that EnvZ-OmpRs are also employed by these chemoautotrophic and acidophilic bacteria to cope with the osmotic stress induced by elevated salinity.

The regulation of membrane permeability by OmpR might be a strategy for *A. caldus* to achieve osmoregulation in a high-salt environment. Unlike OmpR-regulated osmoregulation in heterotrophic neutrophils [19,41], the homologues of the outer membrane porins OmpF and OmpC are absent in these autotrophic and acidophilic bacteria. Acidophiles can regulate the permeability of the cytoplasmic membrane to adapt to osmotic pressure [42,43]. The strong regulating effect of OmpR on membrane and channel proteins (Table S2) confirmed that *A. caldus* employs EnvZ-OmpR to modulate its membrane permeability according to the concentration of ions in the environment. Although chloride salts have a far greater inhibitory effect on *Acidithiobacillus* spp. than do sulfate salts [43], *ΔompR* showed a relatively good growth capacity in 0.45 M NaCl, in contrast to its failure to grow at the same concentration of sulfate salts (Figure S1 and Figure 3). This growth difference suggested that the type of salt has an obvious influence on the adaptation of the *A. caldus ompR*-knockout strain to osmotic stress. In many halophilic archaea, intracellular accumulation of chloride and potassium is an important mechanism for dealing with high osmotic pressure [44]. Thus, the better growth capability of *ΔompR* at high concentrations of NaCl indicate the presence of a similar osmoregulatory mechanism in *A. caldus*. 

Regulation of sulfur oxidation by OmpR may help *A. caldus* adapt to osmotic stress. It has been reported that a high concentration of salt (200 mM) lowers the sulfur oxidation rate of *A. thiooxidans* [12]. Sulfur-metabolic processes in *A*. *caldus* include activation and oxidation of elemental sulfur in the outer membrane, thiosulfate-metabolic pathways in the periplasm, and sulfur-oxidizing enzymes in the cytoplasm. The two periplasmic thiosulfate-metabolic pathways, the tetrathionate intermediate thiosulfate oxidation (S_4_I) pathway and sulfur oxidizing enzyme (Sox) system, were both affected by the absence of OmpR under exposure to high salt (Table S2). The S_4_I pathway consists of a thiosulfate: quinol oxidoreductase (Tqo) and a tetrathionate hydrolase (TetH), which are responsible for oxidizing thiosulfate to tetrathionate and hydrolysing tetrathionate to thiosulfate and other products, respectively [40,45]. As thiosulfate is incompletely oxidized by the Sox system, this compound is considered a joint substrate for the S_4_I and Sox pathways. Tetrathionate in the periplasm is proposed to be transferred by DsrE/TusA into cytoplasm for further oxidation [46]; thus, the concentration of tetrathionate might affect periplasmic and cytoplasmic sulfur-oxidizing processes. Moreover, thiosulfate is unstable at pH < 4, whereas tetrathionate is acid stable [47]. Therefore, S_4_I pathway-mediated conversion between S_2_O_3_^2−^ and S_4_O_6_^2−^, not only influences periplasmic and cytoplasmic sulfur-metabolizing pathways, but also the concentration and species of sulfur substrates in acidic environments. The direct and negative regulation of OmpR on the S_4_I pathway was discovered based on the up-regulation of tetH and tqo in ΔompR and the binding ability of OmpR to the tetH promoter region (Table S2 and Figure 6). This discovery suggests that *A. caldus* invokes EnvZ-OmpR to control the expression of the S_4_I pathway directly, further influencing the expression of other sulfur-oxidizing enzymes and the sulfur-metabolic processes. Thus, the EnvZ-OmpR-mediated regulation of sulfur metabolism might be an adaptation of *A. caldus* to osmotic pressure.

**Figure 7:**
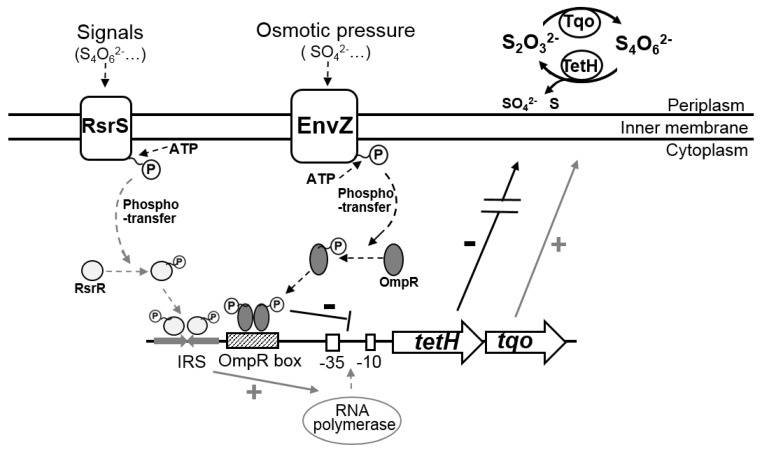
The co-regulation mode for the S_4_I pathway by RsrS-RsrR and EnvZ-OmpR in *A. caldus*.

A co-regulation mode of the S_4_I pathway mediated by EnvZ-OmpR and RsrS-RsrR was discovered in *A. caldus*. A previous study revealed that RsrS-RsrR positively regulates the S_4_I pathway via the binding of RsrR to a 19 bp inverted repeat sequence (IRS) in the *tetH* promoter region [35]. In this study, the direct and negative regulation of the S_4_I pathway by EnvZ-OmpR was revealed for the first time. While RsrS-RsrR is considered an EnvZ-OmpR like two-component system [35,40], the binding sites for the RsrR and OmpR are not identical and show significant distinction in base composition (Figure 6F). Thus, it is speculated that RsrR and OmpR bind to IRS and OmpR boxes to achieve positive and negative regulation of the S_4_I pathway, respectively. Thus, a model for the co-regulation mode for the tetrathionate-metabolic pathway mediated by EnvZ-OmpR and RsrS-RsrR, is proposed to illustrate the significance of this regulation to the osmotic and acidic adaptations of *A. caldus* (Figure 7). Under favorable conditions, *A. caldus* modulates the expression of sulfur-oxidizing gene via positive regulation of the S_4_I pathway by RsrS-RsrR. As sulfur substrates are oxidized and the ion concentration increases in the solution, the environmental stresses of osmolality and acidity emerges, and *A. caldus* readjusts the expression of sulfur-oxidizing genes via RsrS-RsrR-mediated negative regulation of the S_4_I pathway. 

In summary, we demonstrated that EnvZ-OmpR is a fundamental system for *A. caldus* to adapt to high osmotic pressure. OmpR functions as a global regulator to endow *A. caldus* with adaption in environments of high salinity. The discovery of direct and negative regulation of the S_4_I pathway by EnvZ-OmpR reveals the novel regulatory function of EnvZ-OmpR in sulfur-oxidizing bacteria and the significance of sulfur-metabolic regulation in the ability of *A. caldus* to cope with osmotic stress. The discovery of the co-regulation mode for S_4_I pathway mediated EnvZ-OmpR and RsrS/RsrR in *A. caldus* provides new insights into the sulfur-metabolic regulation and environmental adaptation mechanism in these chemoautotrophic sulfur-oxidizing bacteria.

## Figures and Tables

**Table 1:** Strains and plasmids used in the study.

Strains or Plasmids	Genotype or Description	Source or Reference
**Strains**		
*A. caldus*		
MTH-04	Isolated from Tengchong area, Yunnan province, China	[36]
Δ*ompR*	Δ*ompR*	This study
*E. coli*		
DH5α	F^−^_Φ_80d *lacZ*ΔM15Δ*(lacZYA-argF)* U169 *end A1 recA*1 *hsdR*17(*rk*^−^*,mk^+^) supE*44λ*-thi-1 gyr*96 *relA*1 *phoA*	TransGen Biotech Corp., Beijing, China
SM10	*Thr leu hsd recA* Km^r^RP4-2-Tc::Mu	[37]
BL21 (DE3)	F^−^*ompT hdsSB*(Rb^−^mB^−^) *gal dgmmet*(DE3)	TransGen Biotech Corp., Beijing, China
**Plasmids**		
pSDUDI	suicide plasmid; Ap^r^; Km^r^; oriTRP4; multi-cloning sites	[35]
pSDUDI::ompR(UHA + DHA)	suicide plasmid for *ompR* deletion	This study
pSDU1-I-SceI	Cm^r^; mob^+^; Ptac; containing I*-Sce*I gene	[35]
pET-28a	Amp^r^	Novagen Cor.
pET-28a-ompR	Amp^r^, *ompR*	This study

**Table 2:** Primers used in construction of *A. caldus ompR* mutant, OmpR-expressing plasmid, and assays of protein-DNA interactions.

Primer Name	Primer Sequence (5′→3′)
P1-F	ACGCGTCGACATGGGCAAGATGGCAGGACAACG
P1-R	CGGGATCCGATGCGCCCGCGTTTCTGGACGGAC
P2-F	CGGGATCCTCAGGGGCGCTTCCAACCCGGATGA
P2-R	CCCAAGCTTCGGCCTGAATACTCTGGTTCTGGGTG
oriT-F	TACTAGACTAGTGCTCGTCCTGCTTCTCTTCG
oriT-R	ACCGGAATTCCGGGATTCAACCCACTCG
LHA-F	CAAGATGGCAGGACAACGC
LHA-R	GGCGGAGGTGTTCATGGTTA
IHA-F	GGTGCGGGAAGTTTAGGGC
IHA-R	CAAAGGAGAATGCAATGAAAATGTT
ompR-F	GCAGGATTTCAGCGTCTGTG
ompR-R	GACAGGGTCTGCCAGGGAT
ompR_02628-_F	CGCGGATCCATGGCCAAGGCCCGCATC
ompR_02628_-R	CCCAAGCTTTCAGGTGGCCGTGGAGCC
G360-F	CGCAATATTCTGCGGGCGGTC
G360-R	TACGGTAAGGTCCACCGGAT
T360-F	AGCGCCGATTGTGTACAGAATGAAC
T360-R	GATATATAATCTCCGAATCGCTAAT

Restriction sites were indicated with underlines.

**Table 3:** Analysis on the potential OmpR boxes upstream of the DEGs.

Gene ID (F0726_)	Gene	Annotation	Regulation	RNA-Seq	RT-qPCR	Computational Marching of the OmpR Consensus		
						Position	Sequence	Score
1027	*tetH*	tetrathionate hydrolase	-	2.26	3.78	R-122…-103	TTTAGCTTATGTAACAAGTT	13.1
						D-127…-108	GTTACAACTTGTTACATAAG	12.8
						R-132…-113	GTAACAAGTTGTAACAGGTG	8.6
48	*dsbD*	thiol:disulfide interchange protein	-	16.25	24.92	R-128…-109	CCTAACTCAAGAAACATTTT	7.8
1023	*nodT*	RND efflux system, outer membrane lipoprotein, NodT family	-	3.45	2.96	D-405…-386	TTTACGATTTGTTACAAAAA	11.9
						R-410…-391	GTAACAAATCGTAAAATATG	10.7
						R-419…-400	CGTAAAATATGTAACATCCT	9.4
						D-374…-355	TTGACAGATTGTTACACAAC	8.3
						D-424…-405	CTACCAGGATGTTACATATT	7
2394		DEAD/DEAH box helicase domain protein	-	4.35	5.06	R-23…-4	GTTACCGTTGTTTATATTTT	8.4
						D-107…-88	ATAAGTTCTGGAAATAAATA	7.2
3003		TonB-dependent receptor	+	0.29	0.12	R-54…-35	TGTAAGTTTGAATGCAATTT	7.7
						D-387…-368	GTCTAATATTGAAGTATCTC	7.2
2726	*mscS*	mechanosensitive ion channel	+	0.64	0.61	R-54…-35	TTTTAGTCTAGAAACATCGT	10.5
1798		membrane protein	+	0.09	0.07	R-341…-322	TAAAAAGCGAGAAACATCAT	7.5
1192		translation initiation factor IF-2	+	0.56	0.41	D-104…-85	ATAAAAAAATATAACAAGAA	8.8
						R-116…-97	TTTTTTATATAAAACAATTA	8.6
						D-121…-102	CTATATAATTGTTTTATATA	8.4
2316	*motA*	flagellar motor protein	+	0.28	0.32	R-202…-183	TTCACAAATAGTTGCACCAA	7.5
						R-404…-385	CTAAAAAATCGGAACAACTG	7.1
2322	*ade*	adenine deaminase	+	0.32	0.37	D-395…-376	GTCACAGCATGTTACATGTA	10.2
						R-381…-362	AAAAATCTTTGTAGTACATG	8.9
1900		transposase, IS4	+	0.59	0.52	R-407…-388	ATTTGTCTTTGATACTAATT	10.1
						R-466…-447	ATTAATATTGGTTTTATAAG	8.4
						D-492…-473	TAAATATATAGAGATAAATT	7.9
						D-412…-393	TCTACAATTAGTATCAAAGA	7.9
						R-598…-579	AATACAATAACTAGTATTGT	7.7
						R-504…-485	TATATTTATTATTCTATCTT	7.7
1551		ISPsy4, transposition helper protein	+	0.01	0.02	D-73…-54	AAAACTTTAACATACCTTTT	10.3
						R-377…-358	ATAACACTTTATATCCATTC	8.4
2814	*xerC*	Integrase	+	0	0.01	R-470…-451	GTAACTATTTGATTCTTAGA	8.5
						D-155…-136	GAATATTAAGGAAATAGTTT	7.6
						D-465…-446	GAATCAAATAGTTACTGTTC	7.6
1869		transposase	-	3.54	4.21	D-267…-248	AATAAATTTTATGTCATTGC	7.6
						D-576…-557	GATTATGTTCGAATCTTATG	7.2
426		diguanylate cyclase	+	0.3	0.26	D-181…-162	GGAGATCTTTGATACCTTTA	7
2335	*ahpD*	alkylhydroperoxidase like protein	+	0	0	D-493…-474	AAAAAATATTGAAAAATCTC	11
222		AmmeMemoRadiSam system protein B	-	17.11	18.38	R-328…-309	ATTTCATTTAGATGCTTTTG	9.9
						R-453…-434	TTTAAGCTTCGTAATAAAGG	7.7
						D-526…-507	AATAGACTCCGTAACAAAAT	7.5
2436		type II DNA modification enzyme	-	1.82	1.41	D-671…-652	TGTACACTTTGATGTACATA	7.4
2272		hypothetical protein	+	0	0	R-31…-12	TTTATTTTTTATTAGATATA	9.5
2500		hypothetical protein	-	2.56	2.55	R-109…-90	AATACAGATAGTTAGGATTT	7.6
						D-309…-290	TTTACATTCATATACATCAT	7.2
650		hypothetical protein	+	0.63	0.6	D-57…-38	TAAAGACATCGATACAAAAG	8.6
1813		hypothetical protein	-	2.37	3.53	R-628…-609	AGAAATGATGGTTGCAATGT	8.4
1814		hypothetical protein	-	7.31	9.11	R-236…-217	AGAAATGATGGTTGCAATGT	8.4
2504		hypothetical protein	+	0.41	0.33	R-35…-16	ATAAATATTAGAAATATTTC	12
						D-36…-17	CGAAATATTTCTAATATTTA	7.8
1734		hypothetical protein	+	0.07	0.18	D-490…-471	TTTAAATAATGAACCAATGA	8.1

Fold Change ≥ 1.5, *p*-value ≤ 0.05: up-regulation; Fold Change ≤ 0.67, *p*-value ≤ 0.05: down-regulation. The numbers indicate the nucleotide positions upstream of the transcription start sites; -: negative and direct regulation; +: positive and direct regulation.

## Data Availability

The genome sequence of *A. caldus* MTH-04 was deposited in NCBI with GenBank: CP043926.1. The nucleotide sequences of *rsrR* and *rsrS* have been deposited with GenBank accession numbers KX161704 and KX161705, respectively. The raw data of RNA-seq is deposited in NCBI with accession number SRA1121784.

## Supplementary Materials

The following supporting information can be downloaded at: https://www.mdpi.com/article/10.3390/microorganisms11010035/s1, Figure S1: Growth analysis of *ΔompR* and wild-type strains of *A. caldus* at different concentrations of NaCl or K_2_SO_4_ in S0 medium; Figure S2: Bland-Altman plot of differences against averages of fold changes for RNA-seq and RT-qPCR, with 95% confidence limit (the upper and lower solid lines); Table S1: Primers used for RT-qPCR; Table S2: Differentially expressed genes detected by RNA-seq and RT-qPCR.
