# Supplementary material for: The Essential Role of OmpR in Acidithiobacillus caldus Adapting to the High Osmolarity and Its Regulation on the Tetrathionate-Metabolic Pathway
Source: Microorganisms. 2022 Dec 22;11(1):35. doi: 10.3390/microorganisms11010035 (PMC9861516; doi:10.3390/microorganisms11010035)
Supplement: Supplementary file 1 [file microorganisms-11-00035-s001.zip › microorganisms-2081841-supplementary.pdf]

The Essential Role of OmpR in *Acidithiobacillus caldus* Adapting to the High Osmolarity and Its Regulation on the Tetrathionate-Metabolic Pathway

Linxu Chen *,†, Xiao Liu †, Chang Gao, Yanan Guan, Jianqiang Lin, Xiangmei Liu and Xin Pang *

State Key Laboratory of Microbial Technology, Shandong University, Qingdao 266237, China
* Correspondence: linxuchen@sdu.edu.cn (L.C.); pangxin@sdu.edu.cn (X.P.)
† These authors contributed equally to this work.

Table S1. Primers used for RT-qPCR.

Annotation in genome of *A.caldus* MTH-04		Primer Sequence (5'→3')
F0726_00045	F	GGGCAGATTATGGGAGA
	R	CGTCAATTCCGAACCA
F0726_00048	F	ATCGTTATCGGCACCTCAG
	R	CAAGGCAACACCGTAGACC
F0726_00105	F	ACGCTACAAAGGGACCAA
	R	GAGCCCGAAGCATTACC
F0726_00222	F	GCACTGACCTTAGCCATTATCTC
	R	TCATGCCGCATCCTCGTA
F0726_00324	F	GCAGCATCCTGGGTCTTCA
	R	GACCAAAGTGTCTGCCACTGAT
F0726_00426	F	GGGGATCGTCTCACGCTAT
	R	GCAGGATGATGCCAAACTC
F0726_00650	F	TCAGTGAGAAACTTTGGGAGG
	R	ATGGGTTCCACAATCTTCG
F0726_00801	F	TTCACCTTGCCCGAAGAGAC
	R	ACCGCTACGTGGATGTGTTT
F0726_01023	F	CGATGTCTTCGGTGGTCAGG
	R	TGATGGCTTGCTGTTGTTCC
F0726_01027	F	CCAAAGGCAAGGATAAGG
	R	CGAACCATTGAGACACCC
F0726_01192	F	CCTCGTGGACAGGCAACTCA
	R	CCGCAAACGCCAGAAGGT
F0726_01551	F	GGTACTTGCCGTTCTCGG
	R	CATGGCGGTGGTCATCTT
F0726_01733	F	AGTTCCAGGACTTGCTGTTTGT
	R	CGAGGTAATCGGTGGTAAAGG
F0726_01734	F	TACACCAATACCGCCTCC
	R	CAACATCGCCGTTAGAAG
F0726_01798	F	TCTGCGGGTGGTAATAGGA
	R	GGCATAAGCCGGACAAAA

F0726_01813	F	GTTGGATTTCCGTGGGTG
	R	GTGTTGGTTGCGACTGCT
F0726_01814	F	ATGAACGACCGTAACCGTAT
	R	GATCTTCTGACAGACCCCTTT
F0726_02203	F	CGTCGAGGTCAATCATTTAGG
	R	GCCCACTTATTCGGCACA
F0726_02272	F	CATCGCCCATAACTTGC
	R	CTACCGTTGAAACCTGCT
F0726_01900	F	GCAACTCGTAGACCCGTCCTT
	R	CGTGTCCACTATCGGCAGAAAT
F0726_02316	F	GCGTTGCGGCATTTC
	R	GTCACCGAGGCTGGATT
F0726_02322	F	GACAGTTTACCGTTGTTGA
	R	CGCAGTGAAGCCCTAT
F0726_02335	F	TCCGTGCCTCGCAAATC
	R	GCCACCGTCAGAAACACTATC
F0726_02394	F	CAACCCACCCGACATTCT
	R	ACGGCGCACCAACAGA
F0726_02436	F	GCTCAACACCTATCCCTTATTTGC
	R	CTTCATGCGGCTATCCACC
F0726_02439	F	TTACAACCTGCTTTCGCTCCT
	R	CGTTTCCGCCTGCCATT
F0726_02465	F	GAGGAGACCGTCCATGTCA
	R	TGCGGGTGCAGCAGATA
F0726_02500	F	GTCAGCCAGATAGATAGTCCAA
	R	TCCCAGAGCACGACACC
F0726_02503	F	ATGTTCGCATCCCAGTAA
	R	GGAAGTCGCCCAAATAG
F0726_02504	F	CCCAACTTCATTCCCACC
	R	CGCTACCTGTATAGTCTCCTCC
F0726_02628	F	AGGATTTCAGCGTCTGTGGC
	R	TTCGGGCTCGAAGGGTTT
F0726_02726	F	CGGTCTAACCCTGCTCAT
	R	CGACATCGCCATAGTTCA
F0726_02814	F	AGGACGAGCAGGAATATCGG
	R	TGTAGGTGTTGGGCAATGAA
F0726_01869	F	CGAGGGCAAGAAGGAGGT
	R	TGACGGGTTTGTGGGTAAAT
F0726_03003	F	AGATAGCGGGATACAATG
	R	GGCGTAATCGCAACAC
F0726_ 02641	F	ACGTCTCCATCGTCGATCTCA
	R	AGGGCTTGTCGTTGTAGGCA

Table S2. Differentially expressed genes detected by RNA-seq and RT-qPCR.

Gene ID (F0726_)	Gene	Annotation	RNA-seq	RT-qPCR
01027	*tetH*	tetrathionate hydrolase	2.26	3.78±0.02
01028	*tqo*	thiosulfate:quinol oxidase	1.74	
02556	*soxY*	sulfur covalently binding protein	0.62	
02557	*soxZ*	sulfur compound chelating protein	0.57	
02558	*soxB*	sulfate thiol esterase	0.56	
02562	*soxA*	cytochrome c (diheme)	0.58	
00801	*sdo*	sulfur dioxygenase	0.62	0.59±0.01
00103	*narJ*	nitrate reductase chaperone	0.51	
00104	*narH*	nitrate reductase, beta subunit	0.47	
00105	*narG*	nitrate reductase, alpha subunit	0.64	0.61±0.01
00100	*narK*	nitrate/nitrite transporter	0.58	
01243		alcohol dehydrogenase	0.63	
00053	*bcsA*	cellulose synthase, subunit A	0.37	
02707		gamma-glutamyl kinase	0.28	
02923		sucrose-phosphate synthase	0.61	
02503		peptidase C39 family,	0.34	0.32±0.02
02504		hypothetical protein	0.41	0.33±0.01
02505		hypothetical protein	0.25	
02506		hypothetical protein	0.20	
02507		glycosyl transferase	0.16	
00669	*plsX*	glycerol-3-phosphate acyltransferase	1.61	
00877		diacylglycerol kinase	2.82	
02669		methyltransferase	2.05	
01008	*glcE*	glycolate oxidase FAD binding subunit	2.01	
01009	*glcD*	glycolate oxidase, dehydrogenase subunit	1.97	
01010		sugar-binding, N-terminal domain	2.01	
01011		Transketolase, N-terminal subunit	2.01	
00048	*dsbD*	thiol:disulfide interchange protein	16.25	24.92±0.06
01083	*dsbG*	thiol:disulfide interchange protein	0.57	
01826		cytochrome c biogenesis protein	5.66	
01597	*hmp*	flavohemoprotein	0.42	
03003		TonB-dependent receptor	0.29	0.12±0.02
00324	*znuC*	ABC-type Mn2+/Zn2+ transport system, ATPase component	0.46	0.57±0.01
00325	*znuB*-1	ABC-type Mn2+/Zn2+ transport system, permease component	0.41	
00326	*znuB*-2	ABC-type Mn2+/Zn2+ transport system, permease component	0.47	
02726	*mscS*	mechanosensitive ion channel protein	0.64	0.61±0.01
02727		lysogenization protein	0.24	
01013		permease, major facilitator superfamily	1.97	
01023	*nodT*	RND efflux system, outer membrane lipoprotein,NodT family	3.45	2.96±0.03

01798		membrane protein	0.09	0.07±0.02
01812		conserved hypothetical protein	2.78	
01813		conserved hypothetical protein	2.37	3.53±0.04
01814		hypothetical protein	7.31	9.11±0.06
01815		transmembrane protein	8.37	
01816		conserved hypothetical protein	8.56	
01552		membrane protein	0.15	
00169	*qseC*	two-component sensor histidine kinase	0.63	
00914	*rpoE*	RNA polymerase ECF-type sigma factor	0.52	
01192		translation initiation factor IF-2	0.56	0.41±0.01
02418	*abrB*	AbrB family transcriptional regulator	0.61	
02419		PIN domain nuclease	0.56	
02420		WGR domain protein	0.44	
02465		hypothetical protein	0.00	0.00±0.02
02466		transcriptional regulator, ArsR family	0.00	
02627	*envZ*	osmolarity sensor histidine kinase	2.80	
02628	*ompR*	osmolarity response regulator	0.00	0.00±0.02
02199		hypothetical protein	0.01	
02200		hypothetical protein	0.00	
02201	*trbI*	conjugal transfer protein	0.00	
02202	*trbG*	conjugal transfer protein	0.00	
02203	*trbF*	conjugal transfer protein	0.00	0.01±0.02
02389	*traA*	Ti-type conjugative transfer relaxase	0.20	
02390		hypothetical protein	0.12	
01512	*fliQ*	flagellar biosynthetic protein	0.13	
00039	*pilZ*	pilus biosynthesis protein	12.81	
02316	*motA*	flagellar motor protein	0.28	0.32±0.01
02317	*exbD*	biopolymer transporter	0.35	
01732		type I restriction-modification system, specificity subunit S	0.09	
01733		type I restriction-modification system, DNA-methyltransferase subunit M	0.34	0.41±0.01
01734		hypothetical protein	0.07	0.18±0.03
00082		Type I site-specific restriction-modification system, R subunit	1.61	
02436		type II DNA modification enzyme	1.82	1.41±0.02
02298	*csf2*	CRISPR type IV/AFERR-associated protein	0.48	
02372	*cmk*	cytidylate kinase	0.63	
02425		histone-like DNA-binding protein	0.01	
02320	*fcy*	purine-cytosine permease	0.27	
02322	*ade*	adenine deaminase	0.32	0.37±0.01
01246		ribosomal protein L25	1.62	
02439		helicase domain protein	4.17	5.12±0.02
02394		DEAD/DEAH box helicase domain protein	4.35	5.06±0.04
00646		DNA polymerase III subunit epsilon	0.60	

00647		protein of unknown function DUF2237	0.61	
00650		hypothetical protein	0.63	0.60±0.02
01900		transposase, IS4	0.59	0.52±0.01
01551		ISPsy4,transposition helper protein	0.01	0.02±0.05
00021		transposase	0.00	
00242		transposase	0.01	
02814	*xerC*	Integrase	0.00	0.01±0.02
00541	*bet*	phage recombination protein	3.17	
01869		transposase	3.54	4.21±0.03
00110	*dgc*	diguanylate cyclase	0.20	
00426		sensor domain-containing diguanylate cyclase	0.30	0.26±0.05
00427		hypothetical protein	0.18	
02335	*ahpD*	alkylhydroperoxidase like protein	0.00	0.00±0.02
00222		AmmeMemoRadiSam system protein B	17.11	18.38±0.07
00223		AmmeMemoRadiSam system protein A	6.06	
00044		AmmeMemoRadiSam system radical SAM enzyme	3.93	
02272		hypothetical protein	0.00	0.00±0.03
02500		hypothetical protein	2.56	2.55±0.05
00918		hypothetical protein	0.57	
00983		hypothetical protein	0.61	
01197		hypothetical protein	0.39	
02267		hypothetical protein	0.28	
02455		hypothetical protein	0.35	
02933		hypothetical protein	0.26	
02478		hypothetical protein	2.14	
02502		hypothetical protein	3.83	
00045		hypothetical protein	43.06	50.47±0.06
02559		hypothetical protein	0.63	

Fold Change ≥1.5, p-value ≤ 0.05: up-regulation; Fold Change ≤ 0.67, p-value ≤ 0.05: down-regulation.

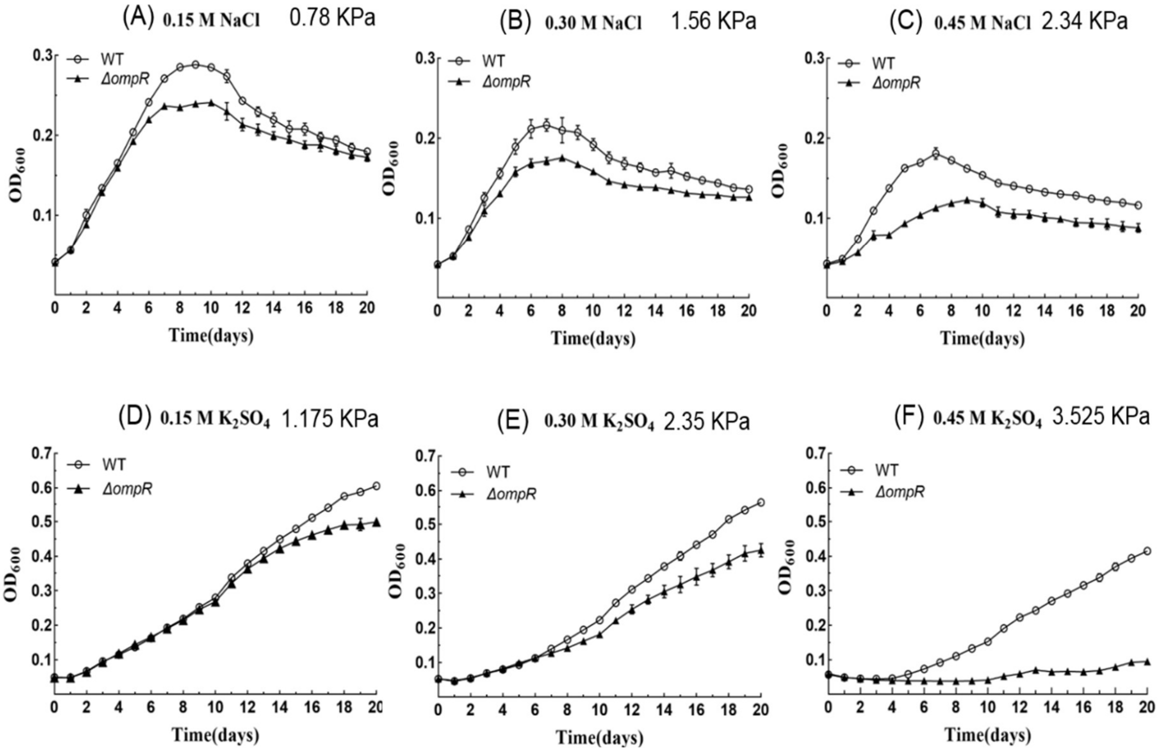

Figure S1. Growth analysis of *ΔompR* and wild-type strains of *A. caldus* at different concentrations of NaCl or K_2SO_4 in S^0 medium.

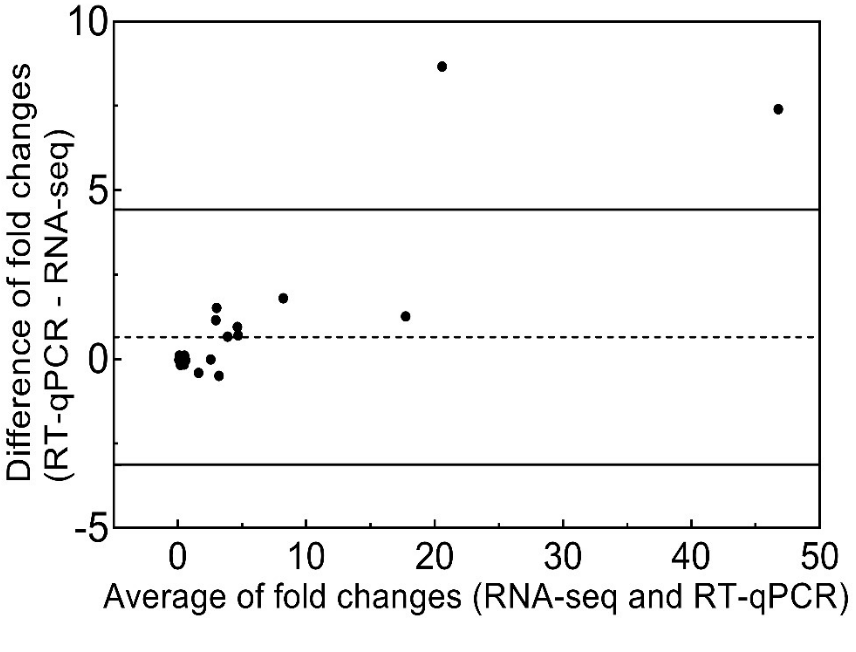

Figure S2. Bland-Altman plot of differences against averages of fold changes for RNA-seq and RT-qPCR, with 95% confidence limit (the upper and lower solid lines).
